# Interprofessional impressions among nursing and pharmacy students: a qualitative study to inform interprofessional education initiatives

**DOI:** 10.1186/s12909-015-0337-y

**Published:** 2015-03-19

**Authors:** Kerry Wilbur, Isabelle Kelly

**Affiliations:** 1 College of Pharmacy, Qatar University, PO Box 2713, Doha, Qatar; 2 Faculty of Nursing, University of Alberta, Alberta, Canada

**Keywords:** Interprofessional education, Pharmacy, Nursing, Middle East

## Abstract

**Background:**

Medical care is increasingly complex and must draw upon the distinct, yet complementary skills of various health disciplines. Healthcare student integration through interprofessional education (IPE) activity is considered one way to promote early, and subsequently sustain, the principles of teamwork. However, It has been demonstrated that each profession has distinct profession-based subcultures, or common attitudes, beliefs and values, even among undergraduate students before commencing their training. We sought to evaluate if undergraduate pharmacy and nursing student in the Middle East had similarly formed attitudes and perceptions of each others’ roles.

**Methods:**

Focus group and semi-structured interviews were conducted with undergraduate pharmacy and nursing students enrolled at Qatar University College of Pharmacy and University of Calgary – Qatar Nursing programs. An eight-question topic guide was developed following comprehensive literature review of reports of other interdisciplinary assessments (either quantitative and qualitative). Working theories were drawn by the two primary investigators based on relevant topic characteristics such as expressed roles and purposes for interacting with one other, patients, and physicians, to develop explanatory constructs for the findings and identify patterns in the data. Qualitative analysis of interviews were supported by NVivo10^**©**^ (QSR International 2013) software.

**Results:**

One shared themes across both health professional groups evolved during data analysis: perceptions of collaborative roles. Discipline specific themes included pharmacist knowledge and visibility (nursing students) and nurses as informants and roles in total patient care (pharmacy students). As expected, students with little or no curricular-based structured experiential training yet largely drew upon personal experiences, whereas senior students, who did have some amount of professional context, often mirrored those that have been found in other studies investigating this interdisciplinary partnership in the clinical setting. Basic understanding of one another’s roles were exhibited, but tended to closely follow traditional scripts that are particularly pervasive in the Middle East.

**Conclusion:**

Concepts arising from our work reinforces the importance of reaching interdisciplinary understanding through assorted formal and informal exposures and can inform ways in which future IPE initiatives can be developed among the various health professional training programs.

## Background

Interprofessional education (IPE) has been described as a convergence of educators and learners from more than 2 health professions who jointly create and foster a collaborative learning environment [1]. The World Health Organization strongly encourages efforts to incorporate IPE into health professional training programs recognizing that patient and population outcomes are improved through multidisciplinary and collaborative care [2]. Integration of health professionals as students through IPE is considered one way to promote early, and subsequently sustain, the principles of teamwork. However, barriers to IPE exist, not least of which includes faculty failure to establish a clear understanding of what competencies should be emphasized and measured in an interprofessional curriculum which may stem from differences in beliefs among the disciplines regarding how clinical work should be undertaken [3].

It has been demonstrated that each profession has distinct profession-based subcultures, or common attitudes, beliefs and values, even among undergraduate students before commencing their training [4,5]. Prior work has found trenchant in-group ratings of academic ability and professional competence among students across 10 professional and social care programs surveyed at the outset of their training; first year nursing, pharmacy, and medical students all perceive themselves as more “caring” than members of the other disciplines [6,7]. Such health professional stereotypes can aversely impact teamwork and ultimately, patient care.

It is currently unknown if these findings reflect student perspectives in a Middle Eastern health professional training environment. Four years ago, a Qatar Interprofessional Health Council comprised of individuals from multidisciplinary academic and practice settings was formed. Its mission was to enable interprofessional collaboration in health care education and practice and subsequently various research projects were launched under its auspices [8]. In order to meaningfully inform development and execution of formal IPE curricular initiatives intended to arise from this work, we sought to explore undergraduate pharmacy and nursing student attitudes and perceptions of each others’ roles in advance of the country’s first multidisciplinary learning activity.

## Methods

A qualitative descriptive study design using focus group interviews for data collection was employed. Undergraduate pharmacy and nursing students enrolled at Qatar University College of Pharmacy (n = 85) and University of Calgary – Qatar Nursing (n = 120) programs were invited to participate. The Canadian-accredited College of Pharmacy is the first and only pharmacy program in the country and has graduated small classes of 5-year Baccalaureate degree pharmacists (female only) annually since 2011. Established in Qatar in 2006, the University of Calgary Nursing program offers regular and post-diploma Bachelor of Nursing degrees to classes of approximately 400 students now each year. Both universities also offer graduate programs in these health disciplines. The student body is drawn from the population of both Qatari nationals and residents of other nationalities born in or living in the country [9,10].

Ethical approval was obtained from the corresponding university institutional review boards. Discipline-specific interviews of consenting subjects were coordinated according to purposeful sampling to include junior (first or second professional year) and senior (third or final professional year) students to capture a range of perspectives based on length of theoretical learning and experiential training. An eight-question topic guide was developed following comprehensive literature review of reports of other interdisciplinary assessments (either quantitative and qualitative). The devised framework sought to explore pharmacy and nursing student experiences, opinions, attitudes and perceptions towards each others’ roles in patient care (Table 1). At the end of each discussion, participants were given the opportunity to ask additional questions or make further contributions. These audio-taped focus group and semi-structured interviews were conducted and transcribed by the professionally-matched primary investigator (IK for nursing, KW for pharmacy) and subsequently verified independently by two separate research assistants.

**Table 1:** **Focus group topic guide questions**

**Engagement Questions:**	
	1. How have you interacted with pharmacists/nurses in your personal life?
	2. How have you interacted with pharmacists/nurses in your professional life?
**Exploration Questions:**	
	3. What do you think are roles of pharmacists/nurses?
	4. In what ways to you think the activities of pharmacists and nurses overlap?
	5. In what ways do you think the activities of pharmacists and nurses are different?
	6. Do you think it is important for pharmacists and nurses to understand each others’ jobs?
	7. What do you think about interacting with pharmacy/nursing students during your training?
**Exit Questions:**	
	8. Is there anything else you would like to say about how you feel about pharmacists/nurses?

**Figure 1:**
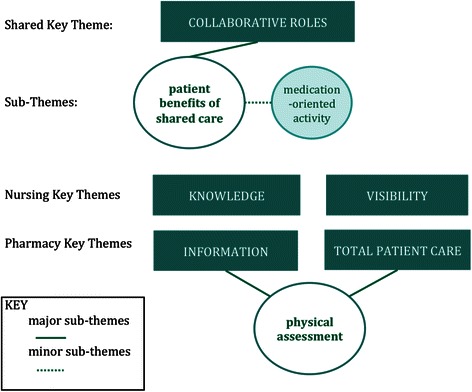
**Conceptual Diagram of Key Themes.**

Qualitative analysis of interviews was supported by NVivo10^**©**^ software. Transcripts were read through several times by primary investigators to obtain the sense of the whole and then subjected to latent content analysis. The text was divided into words, sentences or paragraphs, related to each other through their content and context as units of meaning. The data were then coded and organized around identified sub-themes and themes based on comparisons of their similarities and differences (Figure 1). Working theories were drawn by the two primary investigators based on relevant topic characteristics such as expressed roles and purposes for interacting with one other, patients, and physicians, to develop explanatory constructs for the findings and identify patterns in the data. Respondent validation was undertaken through redistributing a summary of preliminary codes to participants for feedback prior to final theme identification. Inter-rater reliability was achieved by constant comparison, undertaken by primary investigators, and agreement reached [11,12]. Ethic approval was obtained from the Institutional Review Boards of both universities.

## Results

Four facilitated discussions were held between January and March 2013. Nineteen students participated: 9 nursing and 10 pharmacy undergraduates (Table 2). All participants were female. Only the senior students (in both nursing and pharmacy programs) had prior in-patient clinical internship training experiences in their curriculum.

**Table 2:** **Undergraduate Student Participants**

	Junior class	Senior class
Nursing	n = 4	n = 5
Pharmacy	n = 4	n = 6

One shared themes across both health professional groups evolved during data analysis: perceptions of collaborative roles (Figure 1).

### Perceptions of collaborative roles

Both health professional undergraduate cohorts identified distinct tasks fulfilled by the other related to drug therapy products. Junior (Jr) and senior (Sr) nursing students recognized pharmacists in all technical tasks (e.g. steps in drug dispensing) and patient education while pharmacy students acknowledged nursing expertise in medication administration. However, as it pertained to patient-centered drug regimen appropriateness and delivery, students outlined the importance of solving problems together and serving as intermediaries for one another with prescribers. Senior nursing students considered pharmacists as co-adjudicators regarding patient safety issues, as well as accessible resources for clinical opinion.

First, we have to check the doctor’s order with each other. If the dosage is high or low, then we can discuss it with each other, then with the doctor, and then we can come up with options…like if there is a side effect from the medication or the patient develops allergy or we need a generic name for the medication. Then we can discuss it with each other, talk about it. [Sr Nurse]

…and also the doctors are so busy all the time, that if there were a pharmacist there, just like one of the others just said, it would be nice to be able to go and say, “why is this the drug of choice? And the doctor, why did she choose this one?” [Sr Nurse]

Senior pharmacy students also affirmed evidence of a close relationship with nurses.

I think among all the health care providers in the team, the pharmacist and the nurse are the most to communicate and deal with each other. [Sr Pharm]

I observed that nurses like to talk to the clinical pharmacist more than resident or the uh, consultant, in terms of discussing the new stuff for this patient or the changes in the doses. And sometimes nurses suggested uh, they suggest some recommendations for specific patients. [Sr Pharm]

However, there was also concession in terms of how challenges to collaboration arise as pharmacists assume clinical work in new expanded roles within existing domains of nursing care.

I think before the, the uh clinical pharmacist role have appeared, nurses were doing all the inpatient work that the pharmacist are now doing, so this might be kind of overlapping. … the clinical pharmacists here are still not really obligated to do every single thing, so still the role is not really clear. So the nurse might do the discharge counseling while it is the role of the pharmacist, especially if it’s in terms of medications. So, um, now it’s like it, now the role is changing and this is like, we can see a see a clear overlapping; it’s a duplication. [Sr Pharm]

All students identified important aspects and benefits of their complementary roles. Both pharmacy student groups expressed positive attitudes towards the value of nursing contributions to patient care.

Our profession needs us to interact with each other to provide the best service for the patient. If everyone works alone, um, a lot of mistakes, errors could happen. [Jr Pharm]

Patient care would be better if the patient feels comfortable, like the whole team is supporting them, rather than fighting for something. [Sr Pharm]

These positive multidisciplinary sentiments were supported by the nursing student groups.

I think it is important, highly important, that each of them understand their role so they can cooperate together and they can apply the best care for the patient. [Jr Nurse]

### Nursing students

Among the nursing student cohort, two distinct themes recurred throughout the discussions: pharmacist knowledge and visibility (Figure 1).

### Knowledge

While senior nursing students believed they were most knowledgeable about the specific medications they would be administering, all students identified pharmacist expertise related to medication (e.g. chemical content) and informing others (e.g. nurses, physicians, patients) about side effects and drug interactions.

Pharmacy students know the structure of the medication, and what is inside the medication, what would cause this allergy… so that’s very important! [Sr Nurse]

For me, pharmacists, if they are working with nurses in the unit, they can reduce our job, they can do the teaching, like teaching the patient with discharge plan [Jr Nurse]

However, some nursing students contended they offered the most complete patient care.

The nurse is different in a way that she is working with the patient and she keeps monitoring the response of the medication; the pharmacist has the knowledge but they cannot see the response of the patient when they take the medications. [Sr Nurse]

The nurse has more interaction with the patient…has more opportunities for interaction and to see the follow up…whereas the pharmacist really sees the front end, and makes sure the front end is okay. But the nurse sees the front and back end. Does that make sense? Like the follow up, I mean she (nurse) sees the whole process through. The pharmacist does not always get that opportunity. [Jr Nurse]

Yet, this sentiment was not shared by all, as illustrated by disparate comments among junior nursing students.

I think the opposite. I think at home (United Kingdom) I see more. I am talking personal life…when I go in to a pharmacy, I see the pharmacist taking their time with them [elderly patients]…once you are outside the hospital, I think the pharmacist sees [patients] more. [Jr Nurse]

### Visibility

Nursing students across professional years drew upon both experiential and personal experience to convey misgivings about local pharmacist practice.

Some of them here in Doha, do not say much…someone hands you the medication and you pay, that’s all. When I am in Canada, before the pharmacist gives me the medication, he first asks if I had taken it before. [Jr Nurse]

The pharmacist should describe the medication for the patient and tell the patient about the side effects and everything. But in Qatar, they are limited, they just give the medication and that’s it. [Sr Nurse]

Well, I am sure they just don’t hand out medications…. They have to knowmore…[Jr Nurse]

The only time I saw pharmacists was when I had to pick up the patient’s medication downstairs. They are behind the glass window and they all look very busy. [Jr Nurse]

### Pharmacy students

Among the pharmacy student cohort, two distinct themes recurred throughout the discussions: nurse roles as informants, with particular emphasis on expertise in physical assessment, and total patient care (Figure 1).

### Information

Senior students identified nurses as a primary vehicle for patient information in both physical delivery (as a task) and acquisition (as care).

They know each assigned file -the nurse knew everything about the case and they answered all the questions when the team asked. [Sr Pharm]

Moreover, there was high regard afforded nursing organization of clinical work and patient information sharing among themselves.

They don’t just leave the next shift to see what is written in the file. They come together and explain to each other and especially for the nurses in the ICU. [Sr Pharm]

Despite this, some pharmacy students expressed frustration in nurses’ ability to impair their own ability to accomplish data gathering to inform their patient care.

When I and another student went to get some information to do the patient case, they didn’t allow us to take the file or see the, uh, patient profile. They were shouting. And one nurse, um, um, I, I took the file and I asked her before I take the file and then when I was, like searching for the information, she, just come and took it from me [*some laughter and some voicing agreement*, “*yeah*”]. [Sr Pharm]

Indeed, there were a number of comments among the senior cohort displaying attitudes towards nurses as an information recorder and not an equal contributor to decision-making.

We go on multidisciplinary rounds and the nurse, the physician, the pharmacist, and also the resident and consultant. And uh, like usually the nurse brings the file and the physician and pharmacist discuss things. [Sr Pharm]

Similarly, junior pharmacy students emphasized nurses as “data gatherers” as a means to improve physician efficiencies.

The work for the physician is more easy and more fast. Uh, instead of taking all the physical assessment by the doctor, they [nurses] can do it. The patient can enter to the physician with already recorded, for example. [Jr Pharm]

Pharmacy students deferred patient physical assessment to the purview of nurses. Junior pharmacy students in particular emphasized nurses ‘screening tasks’ (e.g. taking vital signs) on behalf of physicians in primary care settings. Discussions revealed while physical assessment skills may be useful for pharmacists to understand, such evaluations largely remain in the domain of nurses or physicians. Conversely, nursing students were supportive of physical assessment activities and unlike the pharmacists, articulated how such activity could provide meaning to their care.

They have to rely on their own data when they make clinical judgments, you know, deciding what medications would be best for the patient…[Jr Nurse]

### Total patient care

Pharmacy students repeatedly expressed the importance of psychosocial supportive roles nurses play in patient care and well-being and the respect they have for this intimacy and associated stresses.

Among the different practitioners in a multidisciplinary team, the nurses are the closest to the patient. [Sr Pharm]

I also think that patients need nurses more. Because when someone is sick in hospital, they need nurse to take care of them, tell him what is, if you are going well or not, what is your medication for today, what to take, what not to take [pause] they feel better with the nurse. [Jr Pharm]

### Interest in IPE

When asked about willingness to participate in future IPE initiatives, participants were generally supportive, citing points about developing greater mutual understanding in patient care roles.

In future, [we] will work with each other so [we] are the ones who are expected to change the practice and have the better communication between the pharmacist and the nurses. [Sr Pharm]

Well the whole medical team: pharmacist, doctors, nurses and auxiliary teams (nurse aides and others), everyone should understand each other’s job….[Jr Nurse]

At the same time, some concern was expressed regarding the opportunity to first establish comfort with the responsibilities and expectations within their own profession in order to permit meaningful interprofessional interactions.

I think that the nursing students should first know their own role because until second year you’re still striving to know what’s basically your role, what you are going to do; and it is not just clinical, you have much more options. So then interacting with pharmacy students, after knowing your own role, would be better. [Sr Nurse]

## Discussion

Our research contributes to the paucity of literature examining nursing and pharmacy practitioner perceptions of one another. Several other investigations of interprofessional perspectives have been conducted, but ours is the first to focus exclusively on this health professional pairing with analysis across different professional years. We found the quantity of current structured shared learning or patient care experiences were few, but that interdisciplinary impressions were still created during all types of interactions, both formal and informal. For example, as expected, the most novice cohorts, with little or no curricular-based structured experiential training yet, largely drew upon personal experiences. Basic understanding of one another’s roles were exhibited, but tended to closely follow traditional scripts: nurses as proximate, caring aids to physicians and pharmacists as knowledgeable dispensers of drugs. Meanwhile, the views of the senior students, who did have some amount of professional context, often mirrored those that have been found in other studies investigating this interdisciplinary partnership in the clinical setting. Canadian nurses felt integration of pharmacists on to the hospital ward improved drug-therapy decision-making, continuity of care and safety [13]. Similar sentiments were found among Swedish nurses; however some felt new collaborative activity was highly time- consuming [14]. Hospital nurses in Pakistan regarded pharmacists as knowledgeable drug information experts and patient educators [15]. Far less literature exists exploring pharmacists perceptions of nurses. When present, the data usually considers pharmacists acceptance and confidence in nurses in prescribing roles, which while in a number of instances positive, may suffer from insufficient understanding of primary care nursing roles [16]. A community-wide consultation to inform curriculum of a new nursing school in Jordan generated positive feedback from pharmacists, who believed the nurse to be technically competent and the only professional taking care of the patient “as a whole” [17].

Our findings also represent the first such evaluation conducted in undergraduate health professionals in the Middle East. Qatar is a gas- and oil-rich Arab emirate of nearly 2 million residents occupying a small peninsula in the Persian Gulf. The country’s population growth over the past decade has been met with expansion of both health and educational services. New hospitals will open over the next several years requiring thousands more health professionals to provide care [18]. To help meet this demand and mitigate reliance on an expatriate workforce, Qatar has launched domestic clinical training programs for nurses and pharmacists, as well as various health care providers, including physicians, nutritionists, and respiratory therapists. As these various curriculums emerge, a unique opportunity presents itself to not only assess and ‘intervene’ at this early stage of health education development, but to critically evaluate how planned transplantation of IPE from ‘Western’ curriculums into other cultures take into account issues including the context of beliefs, practices, and needs of its Arab Gulf-based students and its Arab/Muslim patient base [19].

It is through this Middle Eastern context that other observations may be considered. Pharmacy students consistently expressed high respect for nurse proximity to patients, but offered underestimations of other care contributions and knowledge. Unfortunately, this likely reflects a wider negative bias nurses consistently encounter in the region [20]. Prior local work has described practice obstacles faced by a sample of 50 Qatari nurses, not least of which included community and family resistance due to perceived menial, low status position of nurses [21]. In a recent study, Bahrain nursing students outlined societal and cultural barriers they met in pursuing their chosen profession, including an unfavourable public image, but themselves believed a nursing career to be “humanitarian” in nature [22]. Similarly, nursing students believed Qatar pharmacists lacked presence in hospitals and especially community settings (with which many had had positive prior experience in other countries) and is indicative of general low pharmacist expectations in the country. The Qatar public has poor understanding of pharmacist roles, which may stem from insufficient opportunity for meaningful interaction and concerns regarding their knowledge-base [23]. Despite this, there is already evidence of recognition of a professional alliance. Both pharmacy and nursing students invoked instances whereby they served as one another’s intermediaries with physicians. While this is a not unique phenomenon, it can be especially beneficial in this region where deference to the medical authority- and physician expectation thereof-by patients and health team members is entrenched in the practice environment [19]. These students will have to work together to promote the model of patient-centered multidisciplinary care and should benefit from national initiatives in this regard [8].

Adoption of IPE early in health professional training could play a role in such enterprise. Indeed, as evidenced in the formation of a Qatar Interprofessional Health Council, various associated research projects and the enthusiasm among our small student sample, the country is embracing the international proliferation of IPE initiatives [24]. Collaborative learning in controlled simulated settings can promote recognition of complementary skills and expertise, before stereotypes take root. Students in our study identified how they could learn from one another and appreciated how this could favourably impact patient care. Yet work is still required to determine the best timing and format for such integration. A few nursing students astutely expressed concern that poorly-timed or aggressive immersion into IPE activities could dilute their ability to first form their own professional identify. To do so, curriculums in each discipline must offer sufficient opportunities for students to first interact, both formally and informally, with their own members and to explore and even challenge accepted frameworks of established roles [25,26]. Indeed, these findings and lessons from our subsequent initial undertaking of IPE activities among health professional students in Qatar have further underscored the importance of developing collaborations with learning objectives and assessments oriented to student demonstration of competencies in role clarification, interprofessional communication, and shared-decision making with less emphasis on evaluation of specific disease and drug-based knowledge [27,28].

The interpretation of our results is subject to the limitations of all small scale qualitative work; generalizing findings rests on theoretical, rather than, statistical inferences. Our participants are from a single geographical area and cannot be assumed to represent similar target populations regionally or otherwise. However, we feel that the concepts arising from our work reinforces the importance of reaching interdisciplinary understanding through assorted formal and informal exposures and has informed ways in which IPE initiatives can be developed among the various health professional training programs in Qatar and the Middle East region.

## Conclusion

Our research has identified local nursing and pharmacy student viewpoints that can help shape how interprofessional understanding and respect can be enhanced to advance IPE curricular initiatives and patient-centered multidisciplinary care in Qatar and elsewhere.
